# Navigating tumour microenvironment in endometrial carcinoma: a comprehensive review integrating immunohistochemistry, single-cell RNA-sequencing and spatial transcriptomics

**DOI:** 10.3389/fonc.2025.1685565

**Published:** 2025-11-12

**Authors:** Oishee Mondal, Blessy Kiruba, Sajitha Lulu Sudhakaran, Vino Sundararajan

**Affiliations:** Integrative Multiomics Lab, School of Bio Sciences and Technology, Vellore Institute of Technology, Vellore, Tamil Nadu, India

**Keywords:** tumour microenvironment, single-cell RNA sequencing, spatial transcriptomics, immunohistochemistry, endometrial cancer

## Abstract

Endometrial cancer is regarded as one of the most prevalent malignancies in women globally. Despite the advancements brought by multiple therapeutic strategies, the efficacy and effectiveness of treatment still appear to be diminished. Therefore, this accentuates the importance of understanding the tumour microenvironment (TME). Conventional methods, including immunohistochemistry (IHC), along with recently emerged techniques like single-cell RNA sequencing (scRNA-seq) and spatial transcriptomics (ST), have rejuvenated the notion of deciphering TME. From deconstructing tumour heterogeneity, identifying cell populations that play significant roles in treatment response, to discovering key biomarkers and therapeutic targets, these technologies lay the base for innovations. Importantly, this is the first comprehensive review that brings together scRNA-seq, ST, and IHC in Endometrial cancer. Merging these methods is geared towards creating a better grasp of the dynamics and interactions between diverse cells and the TME.

## 1 Introduction

Endometrial cancer (EC) is ranked sixth in the global hierarchy of cancerous tumours (1). The rate of Endometrial cancer increases with age, and the highest reported rate in women belongs to the age group of 75-79 (2). As of the 2022 update, 420,368 new cases and causing 97,723 fatalities. North America has the highest burden of diseases, and subsequently Eastern and Central Europe. There is a rapid rise in global incidence, with about 60% more yearly cases expected globally by 2050. Furthermore, EC is influenced by several risk factors, including reproductive, lifestyle, metabolic, and genetic factors. Among reproductive factors, high lifetime exposure to oestrogen, such as late responsiveness, anovulatory cycles, and nulliparity, significantly increases risks. Obesity is recognized as a significant risk factor, with each 5 kg/m² increase in body mass index associated with an approximately 60% higher risk. Metabolic risk factors such as type 2 diabetes mellitus and polycystic ovary syndrome also contribute to elevated EC risk. From a genetic point, Lynch syndrome, caused by germline pathogenic variants in DNA mismatch repair genes, confers a 13%–49% lifetime risk of EC, and inherited BRCA1/2 mutations are also associated with EC risk (3). In 2013, four molecular subtypes were identified by The Cancer Genome Atlas (TCGA). These include DNA Polymerase epsilon (POLEmut) ultramutated, microsatellite instability (MSI) hypermutated, and copy number high and low. Further, to simplify, the Proactive Molecular Risk Classifier for Endometrial Cancer (ProMisE) framework categorized endometrial tumours into four molecular subtypes: mismatch repair–deficient (MMRd), p53-abnormal (p53abn), no specific molecular profile (NSMP), and POLE ultramutated (POLEmut) (4). Additionally, the FIGO released their most recent staging update for EC in 2023, officially titled FIGO Staging of Endometrial Cancer: 2023 (5). This aids in better understanding the complex nature of the cancer and tumour patterns. This also helps in the enhancement of tailored implementation of precision medicine and risk stratification.

Typically, the early diagnosis of EC in women is characterized by postmenopausal bleeding (6). For Cases of EC confined to stage I or II, the clinical trajectory permits a therapeutic approach restricted to surgical extirpation, with adjunction of brachytherapy or radiation (7, 8). However, the traditional treatment of advanced EC includes a combination of paclitaxel and carboplatin, leading to a median overall survival of 37 months and a 52% objective response rate. It is noticed that half of the patients observe the progress of their disease in the first year of treatment, and further rounds of chemotherapy provide limited effectiveness (9). Taking note of all the challenges, immunotherapy has emerged to be promising. At present, it has been a modality of revolutionary treatment, leveraging the immune profile and its ability to inhibit tumour progression, thus proclaiming new avenues for disease management. Unfortunately, the efficacy of immunotherapy is compromised due to the presence of underlying mechanisms, giving rise to the manifestation of resistance in patients. These mechanisms include genetics and epigenetic alterations within the tumour cells that modulate immune checkpoint molecules, allowing the elusion of immune surveillance (10). Acknowledging these impediments, therapeutic strategies can only be effective if the tumour microenvironment is given prime importance.

**Figure 1:**
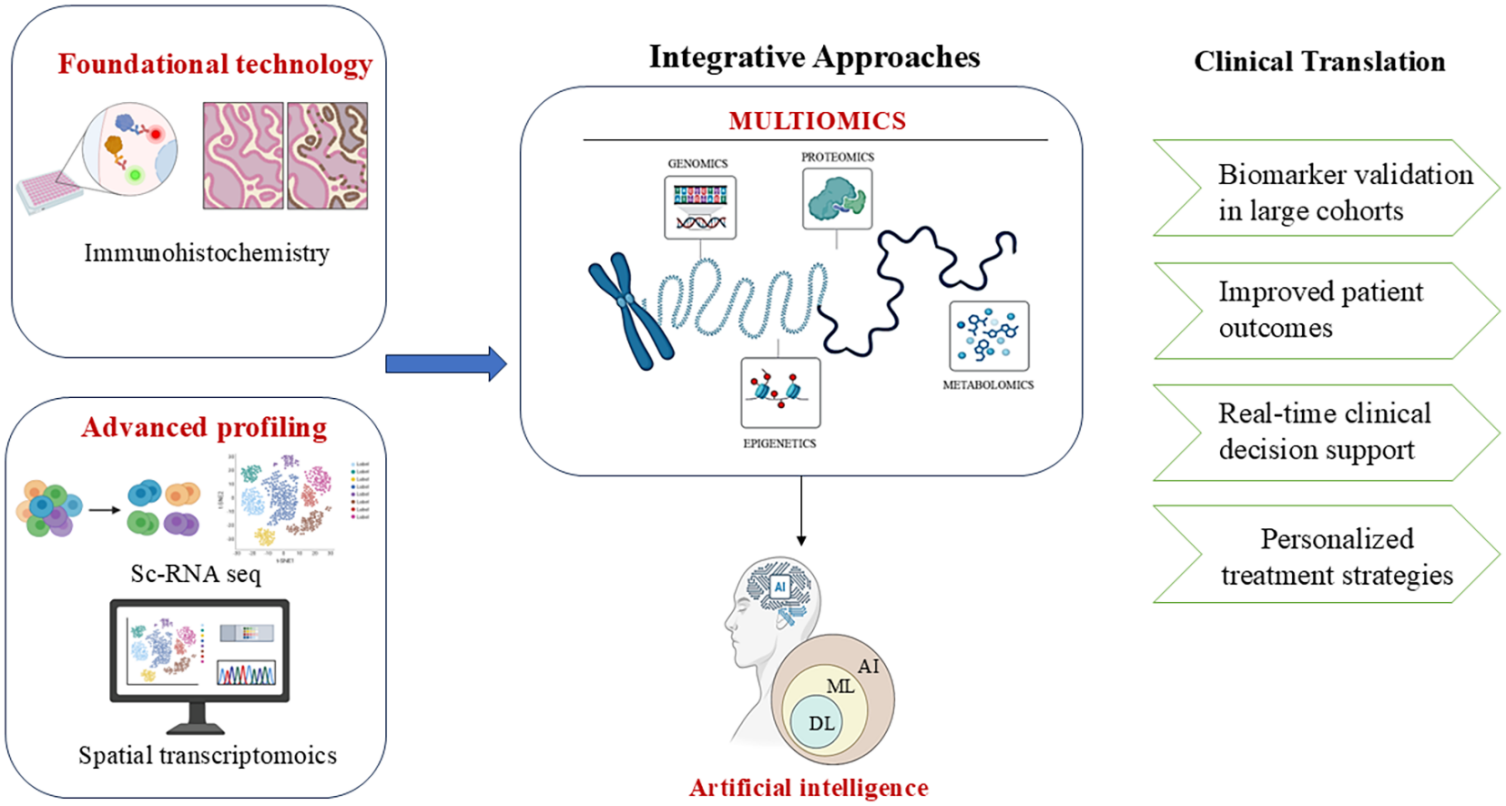
A conceptual roadmap for endometrial cancer research combining AI, multi-omics, and clinical validation. Image created by BioRender (13).

Tumour microenvironment (TME), comprises an elaborate assembly of immune cells, mesenchymal cells, inflammatory mediators, endothelial cells, and extracellular matrix, which surrounds the tumour and facilitates interactions, resembling an ecosystem. Various techniques are available to facilitate the study of TME, each offering a distinct set of perspectives. Recognition of different cell types and evaluation of the gene expression profiles at the single-cell level is aided by single-cell RNA sequencing (11). The excellence of visualization and quantification of proteins in tissue samples is achieved by Immunohistochemistry (IHC). In parallel, spatial transcriptomics integrates gene expression data with spatial organization, such that it becomes possible for researchers to precisely locate various cell types and elucidate their interactions within the tissue structure (12). The cosmic interplay among the malignant, stromal, and immunological factors is a critical parameter influencing the design and optimization of accurate and specific prototypes. Therefore, the rise of these sophisticated methodologies has unveiled such cross-talks. This review focuses on providing a profile of various known and novel methodologies utilized to explore TME in EC. A roadmap of Endometrial cancer research by integrating AI and multi-omics approaches has been highlighted in **Figure 1**. Methods like single-cell RNA sequencing, spatial transcriptomics, and immunohistochemistry are core pillars of this review paper, focusing on the TME and its significance in the formulation of medication for EC patients.

## 2 Immunohistochemistry

**Figure 2:**
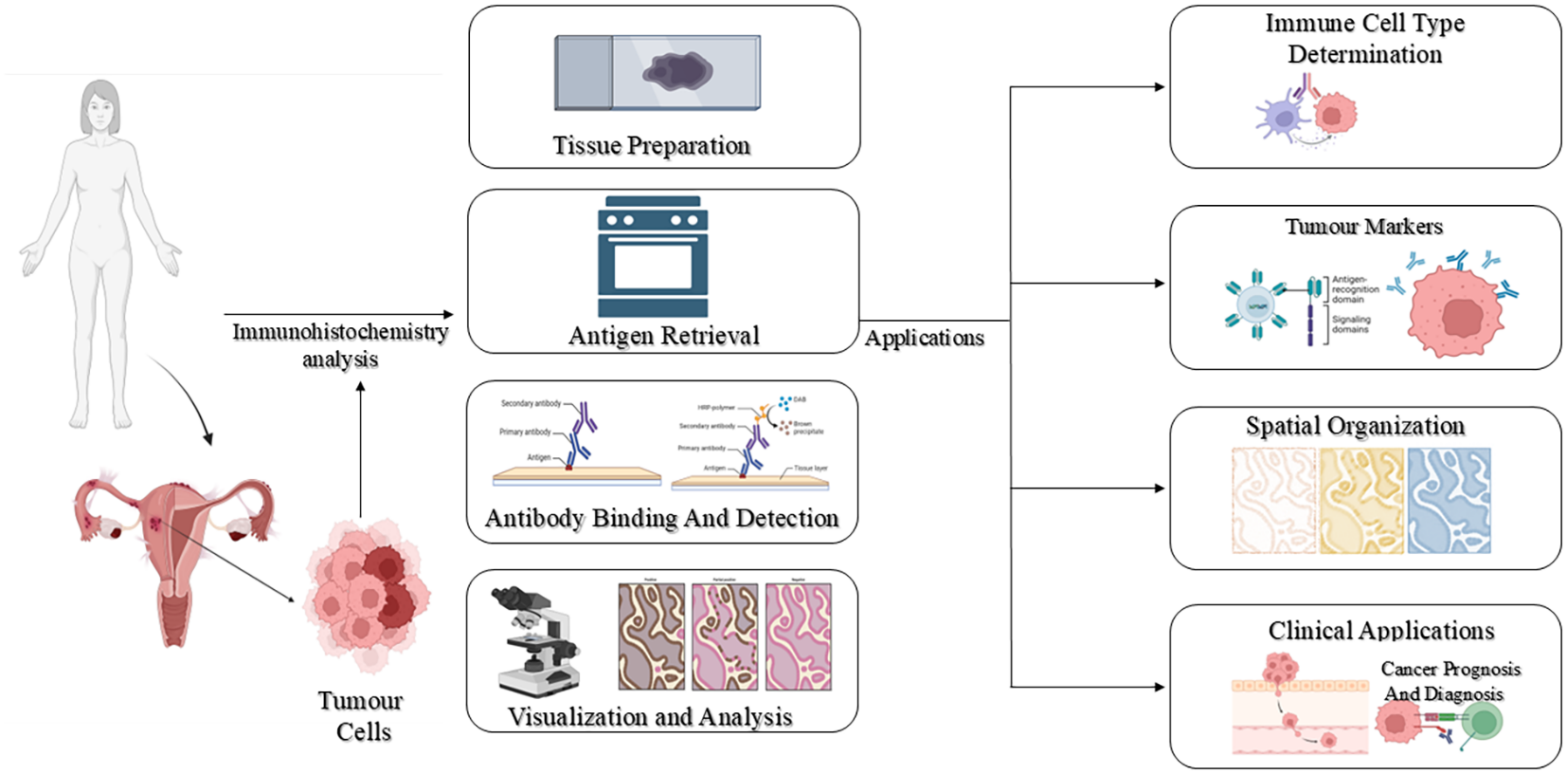
An overview of immunohistochemistry. Image created by BioRender (13).

Immunohistochemistry (IHC) is a sensitive and specific diagnostic method that allows visualization of target antigens within tissue sections by antibody-antigen interactions. It is widely used over a broad range of biomedical applications, ranging from infectious disease diagnosis (14), neoplasms (15), neurodegenerative diseases (16) and myopathies (17). Through the combination of molecular specificity with tissue architecture preservation, IHC delivers spatially resolved, accurate information on the localization, distribution, and quantity of proteins in their native histological environment. The protocol of IHC usually consists of antigen retrieval, incubation with a primary antibody directed against the targeted antigen, addition of a labelled secondary antibody, and lastly, visualization of the localized primary antibody through enzymatic or fluorescent reagents (18). An overview of immunohistochemistry has been depicted in **Figure 2**.

In cancer, IHC revolutionized diagnostic and prognostic routines (19). It facilitates the detection of tumour-specific proteins, differentiation between benign and malignant lesions, and classification of cancer subtypes and stages. Apart from diagnosis, IHC helps in the evaluation of drug action by uncovering changes in therapeutic targets and downstream signalling molecules (20). Using particular histochemical markers, it helps in tumour grade and stage determination and assists cell-type deconvolution in complicated tissue microenvironments (17, 21). In the TME, where heterogeneous immune, stromal, and malignant cells interact with each other, it further plays a role in differentiating among cell populations expressing identical proteins according to their spatial distribution and density (22, 23).

### 2.1 The impact of immunohistochemistry on endometrial cancer

In endometrial cancer, IHC is still among the most commonly used methods for evaluating tumour biology. In contrast to transcriptomic methods that give expression information without spatial reference, IHC provides direct visualization of protein localization and abundance within tissue sections. Thus, it maintains the architectural integrity of the tissue as well as that of the surrounding microenvironment – enabling correlation of molecular alterations with tissue morphology (24). To explore whether molecular types have an impact on tumour morphology, a study was conducted to analyse immune contexture in a cohort of primary, untreated EC, correlating morphological data with TCGA-defined molecular subgroups (POLE-mutant, p53 mutant, MSI, and NSMP). The study revealed MSI tumours with MLH1/PMS2 defects and high-grade POLE-wildtype/MSS tumours expressing high immunogenicity characterized by intense T-cell infiltration, especially at the invasive edge. High density of regulatory T cells (Tregs) was a prognostic indicator of poor outcome in p53-mutant EC, whereas WHO grade continued to be the primary prognostic factor in NSMP EC. In conclusion, the combination of molecular subtype, morphology, and immune context provides a more accurate insight into EC behaviour and prognosis (25). The TME plays a vital role in tumour formation and prognosis. It is also equally responsible for metastasis. To improve diagnostic precision, staging, and disease management, an updated version of EC staging was released by FIGO in 2023 (26). The revised staging incorporated molecular insights into its framework, which was enhanced by IHC-based key biomarkers, particularly mismatch repair (MMR) proteins and p53, forming the base of a better stratification approach (27, 28). **Table 1** depicts the Histo-molecular classification of EC.

**Table 1:** Histo-molecular classification of EC.

Molecular subtype	Frequency and clinicopathologic features	Morphological and molecular characteristics	Prognosis/outcome	References
POLE-mutated EC	POLE mutations are more frequent in high-grade ECs (12.1%) than in low-grade ECs (6.2%) and occur in 12.4% of undifferentiated/dedifferentiated, 3.8% of clear, and 5.3% of carcinosarcomas.	Characterized by nuclear atypia, pleomorphism, heterogeneity, and giant anaplastic cells. Often exhibiting ultra-mutation with high TMB and increased immune infiltration.	Excellent prognosis, reflecting their strong immune response and high mutation load.	(27, 29–31)
MMR-deficient EC	More prevalent in the lower uterine segment and endometrioid histology, with increased incidence in younger patients and those with Lynch syndrome.	Displays microsatellite instability and loss of expression of MMR proteins (MLH1, MSH2, MSH6, PMS2). Histologically, these tumours often exhibit TLS and solid growth patterns.	Intermediate prognosis; MSI-H and MMRd status predict response to immune checkpoint inhibitors.	(28–30)
p53-abnormal EC	Accounts for approximately 20–25% of ECs, most common in serous and high-grade endometrioid subtypes. Frequently associated with older age and advanced stage at diagnosis.	Shows strong diffuse nuclear p53 staining or complete absence (null pattern), often accompanied by chromosomal instability. Frequently co-occurs with copy number alterations and TP53 mutations.	Represents the poorest prognostic group, accompanied by aggressive behaviour, frequent recurrences, and poor survival outcomes.	(27, 28, 30)
NSMP EC (No Specific Molecular Profile)	Accounts for approximately 40-50% ECs, mainly low-grade endometroid; lacks POLE mutations, MMR deficiency, and p53 abnormalities.	Shows frequent mutations in PTEN, PIK3CA, KRAS, and ARID1A. Presents glandular architecture, low mitotic index, and mild nuclear atypia.	Exhibits variable prognosis; intermediate outcomes depending on tumour grade, stage, and L1CAM expression.	(27, 29, 30, 32)

In addition to the canonical markers, IHC has also played a crucial role in assessing other molecular characteristics, such as microsatellite instability (MSI-H), epithelial-to-mesenchymal transition (EMT) markers (33), and hormonal receptors to oestrogen (34). All these assessments together enhance diagnostic accuracy, shed light on disease mechanisms, and guide patient-specific therapy (35, 36). Based on the diagnostic groundwork, current investigations have broadened the application of IHC beyond protein detection to tumour-immune dynamics of the microenvironment. Subsequent evidence has extended this basis to demonstrate a correlation between TLS presence and an activated immune microenvironment. IHC analysis revealed TLSs both intratumorally and in the stroma—consisting of a mixed population of CD8^+^ and CD4^+^ T cells, CD38^+^ plasma cells, and germinal centres (37). In a growing body of research, IHC also revealed that TIL-rich tumours had more CD8+ T cells, whereas immune-exclusion tumours had fewer CD8+ T cells (38). Another study stated that, in T1 stage and G1 grade tumours, elevated levels of stromal TILs suggest their potential as a prognostic indicator for early-stage, less aggressive EC (39). Apart from the determination of immune infiltration, IHC has given critical information regarding immunogenicity and responsiveness to therapy (40). Identification of immune checkpoint proteins PD-1 and PD-L1 by IHC is a very useful measure of tumour immunogenicity and also predictive of the potential benefit from checkpoint inhibitor treatment. As an example, in a study combining next-generation sequencing with IHC, tumour mutation burden (TMB) was measured together with PD-L1, MMR, and TIL expression. PD-1 expression was found predominantly at the tumour–immune interface, highlighting its function in regulating immune evasion and TME remodelling (35). CXCR4, which is involved in tumour invasion, was studied for its interaction with cancer-associated fibroblasts (CAFs) within the TME. 71 ECs (14 endometrial, 57 myo-invasive), 6 EINs (Endometrial intraepithelial neoplasia), and 42 non-neoplastic samples were analysed for the expression of CXCR4 by IHC, and its expression was noted in the cytoplasm and cell membrane (41). Likewise, overexpression of transcription factor GRHL1 and metabolic enzyme glutaminase, both found to be identified by IHC analyses, were significantly higher in EC tissues than normal endometrium, associating these proteins with tumour proliferation and metabolic remodelling (42). In addition, HER2 expression analysis by IHC identified incomplete membranous and basolateral “U-shaped” staining patterns characteristic of the serous component of mixed-type uterine serous carcinomas, which demonstrated the heterogeneity of HER2 signalling in EC subtypes (43, 44). So far, implementing IHC has eased the detection of different protein expressions. The method has illuminated the underlying mechanisms responsible for the disease development and proliferation, ultimately strengthening our understanding of TME. In addition, cervical smear specimens examined by IHC and DNA-based techniques showed abnormal p53 and MSH2/MSH6 expression, with simultaneous gene changes in PTEN, ARID1A, PIK3CA, and TP53—highlighting the possibility of using minimally invasive molecular profiling of EC (45).

### 2.2 Support for computational studies

In modern EC research, IHC has moved from being a merely diagnostic tool to being a critical validation platform for computational and high-throughput investigations of the TME. Staining tissue samples with antibodies specific to various proteins can facilitate the visualization and quantification of immune cell infiltration, expression of immune checkpoints, and other key features. This experimental data can be directly compared to computational studies, adding value to the accuracy and reliability of the computational analysis (46). For example, IHC confirmation helped to validate the computationally predicted MCM10 in EC progression—demonstrating high concordance between the elevated protein expression and tumour invasiveness. The findings indicate that MCM10 serves as a poor prognostic marker in EC, as its overexpression is associated with tumour progression, aggressive clinicopathological features, and reduced overall survival, suggesting that MCM10 may promote EC development and could be a potential therapeutic target (47). Similarly, the functional significance of Myelin and lymphocyte protein (MAL) within the context of EC remained largely unaddressed. Hence, a computational study (differentially expressed genes, GSVA) was performed, and IHC was employed to validate the study. Sequential probing using primary anti-MAL antibody, then a secondary antibody, indicated a positive correlation between MAL expression and advanced histological grade (48). Likewise, IHC validated the overexpression of immune checkpoint protein B7-H4 in various solid cancers, such as EC. The SGN-B7H4V antibody–drug conjugate exhibited strong antitumor activity *in vitro* and *in vivo*, which underscores the translational relevance of IHC-validated targets (49). Furthermore, integrated genomic and IHC analyses of proteins including ARID1A, CD3, CD8, and MMR showed that deficiencies in DNA repair and high levels of TILs, frequently correlated with low ARID1A expression, might be the basis for the good prognosis reported in some EC subtypes (50). Together, these results depict IHC as a foundation technique not just for prognostic and diagnostic classification but also to combine molecular, spatial, and computational information. Its ability to associate cellular localization with molecular information still bridges conventional pathology with next-generation omics, paving the way for accurate immunophenotyping and individualized therapeutic design in endometrial cancer. The clinical relevance of key immunohistochemical markers in EC is tabulated in **Table 2**.

**Table 2:** Key immunohistochemical markers and their clinical relevance in EC.

Markers	Type/significance of marker	Reference
p53, oestrogen	Used for molecular classification and subtyping of EC	(25, 36)
Elevated stromal TILs	Indicator of early-stage EC and favourable immune response	(39)
PD-1 expression, and TILs abundance	Predictive markers for immunotherapy responsiveness	(51, 52)
CXCR4	Associated with tumour invasion and metastasis	(41)
GRHL1	Highly expressed in EC tissues; potential diagnostic biomarker	(42)
HER2	Overexpressed in the serous subtype of EC	(43)
CD8+ T cells	Enriched in TIL-rich tumours	(38)
MSH2/MSH6, PTEN, ADRIDIA, PIK3CA, and TP53	Frequently mutated or altered genes in EC; used for molecular classification	(45)
MCM10	Associated with tumour progression and invasion	(47)
Myelin and lymphocyte protein	Highly expressed in the advanced histological grade of EC	(48)
B7-H4 protein	Overexpressed in EC	(49)
ARID1A	Reduced expression is associated with a favourable prognosis and improved patient outcomes	(50)
Mismatch Repair Proteins (MLH1, PMS2, MSH2, MSH6)	Loss indicates MMR deficiency (MMRd/MSI-H), guiding Lynch Syndrome screening and immunotherapy eligibility	(53, 54)

However, as EC research progresses toward greater resolution of tumour ecosystems, there is an urgent need to overcome the analytical constraints of IHC. While IHC provides insightful single-marker and low-plex information, it does not possess the resolution to elucidate transcriptomic heterogeneity, cellular plasticity, and intercellular communication that characterize the EC TME. To solve this, researchers have increasingly relied on single-cell RNA sequencing (scRNA-seq) and spatial transcriptomics (ST)—technologies with the ability to profile thousands of genes at the resolution of single cells while maintaining spatial context. Next-generation tools offer an unprecedented ability to analyse cellular heterogeneity, map lineage evolution, and define molecular pathways within the tissues’ native environment. Here, IHC has become a confirmatory and complementary method of validation, confirming protein-level expression and spatial localization of primary targets delineated by scRNA-seq and ST analysis (55–57). In combination, they constitute a synergistic platform that couples spatial resolution with molecular depth, making holistic insight into endometrial cancer microenvironment and translational relevance to personalized medicine.

## 3 Single-cell RNA sequencing

The inherent complexity of quantitative analysis in biological systems is unavoidable. The human body is made of a diverse multitude of cells marked by its dynamic transitions, experiencing development, replenishment, and malfunctions (58). A comprehensive catalogue of all the RNA transcripts in a tissue or cell is known as a transcriptome, which gives a snapshot of the gene expression profile at a particular physiological state (59). Since its inception in 2009, scRNA-seq has transformed transcriptomic analysis by offering a previously unattainable resolution of cellular heterogeneity (60). In contrast to the conventional bulk RNA sequencing or microarray methodologies that veil the contributions of distinct or rare populations (61), scRNA-seq molecularly dissects gene expressions at cellular level (62, 63). Hence, scRNA-seq overcomes the constraints of traditional sequencing technologies, which only record average gene expression levels across whole cell populations. This high-throughput, multidimensional method aids in a nuanced knowledge of cellular variety and functionality, resulting in substantial advances in many areas of biological study (64, 65).

**Figure 3:**
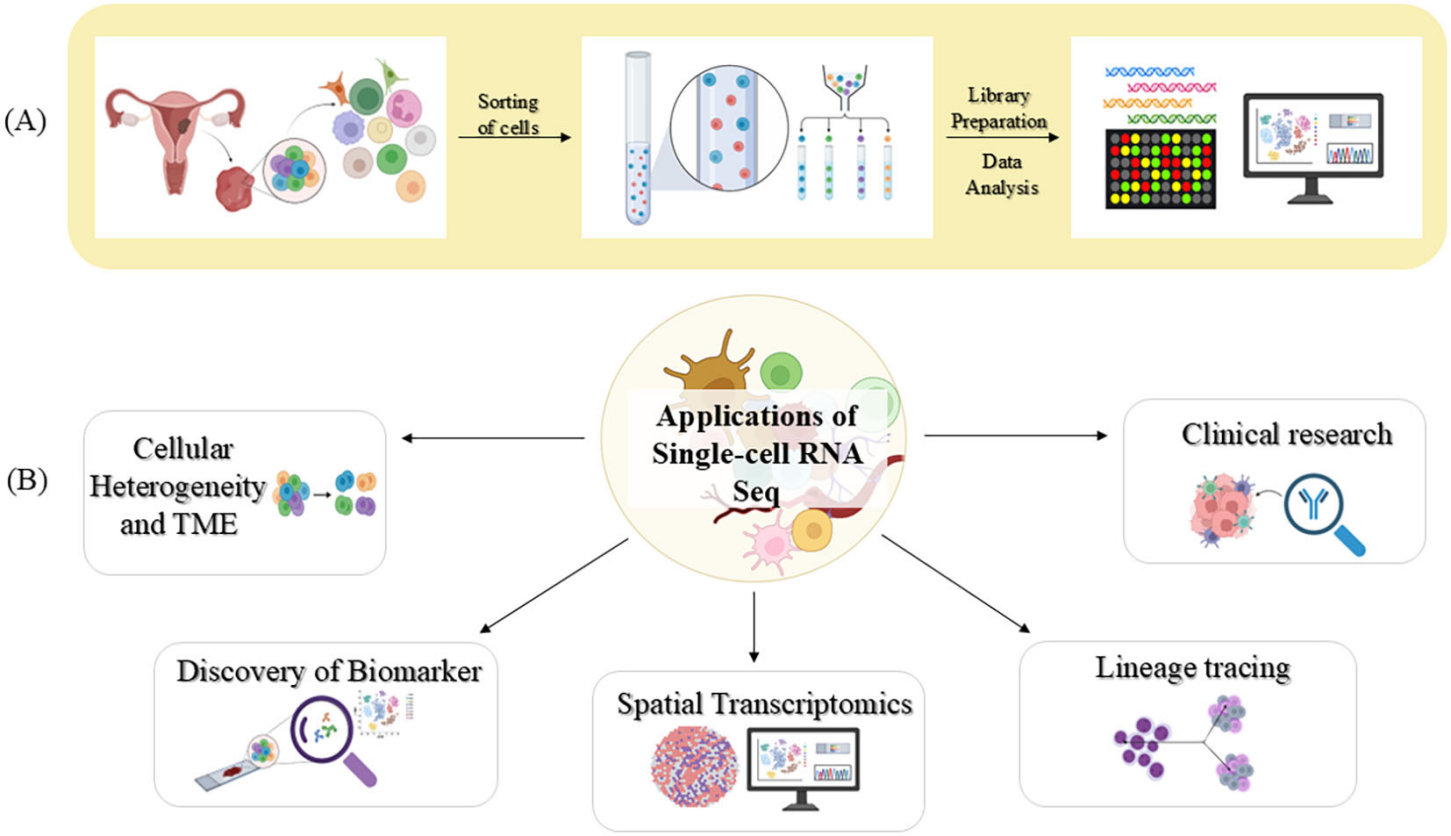
A visual representation of scRNA-seq technique and its contribution in biomedical research **(A)** Workflow of scRNA-seq **(B)** Key applications of scRNA-seq. Image created by BioRender (13).

The preliminary steps in the workflow of scRNA-seq are demonstrated in **Figure 3**. It includes a number of key steps: cell isolation, reverse transcription, and synthesis of cDNA, high-throughput sequencing, and analysis of the data to elucidate the gene expression patterns (65, 66). Every cell has its barcode; hence, combining cells of varying samples does not confuse the process. This allows researchers to analyse several samples at one go on the same sequencing lanes (67, 68). By dissecting the intricate cellular profile, this technology has revealed invaluable insights into cellular composition and its crosstalk in humans, plants, and model organisms (69–71).

One of the most common diseases where cellular heterogeneity plays an important role is cancer. The disease complexity is characterized by cellular transformation and clonal heterogeneity (62). The complicated network of cellular ecosystems in the tumour microenvironment is the driving force for invasion. Advancements in sequencing technologies have empowered the generation of vast molecular data from individual cancer specimens, ushering in the era of precision medicine in oncology. However, pursuing precision medicine demands a patient’s detailed molecular profile, and as mentioned earlier, bulk RNA sequencing struggles to capture the diversity of the TME (67, 72), making scRNA-seq vital for cancer research (73–75).

### 3.1 The ascendancy of scRNA-seq in endometrial cancer

Single-cell analysis offers a high-resolution map of the TME, making it possible to identify actionable targets, direct therapeutic approaches, and enhance the predictive power of clinical trial outcomes (76, 77). Based on this ideology, single-cell transcriptomic profiling has revealed the astounding cellular heterogeneity of EC, showing that the tumour is more than a homogenous mass of malignant epithelial cells but an ecosystem of interacting cell populations. A study of 30,780 cells from tumour and para-tumour tissues around the tumour demarcated a high-resolution cellular atlas of EC and identified seven lymphoid, seven myeloid, and three epithelial cell subtypes. Within the epithelial compartment, three more subtypes were found —stem-like, secretory glandular, and ciliated cells (78). ScRNA-seq technology has also helped in characterizing NK cell heterogeneity in EC, and in a study has successfully highlighted the involvement of CD56dim and DNAJB1, promoting dysfunctionality. CD56dim NK cells represent the predominant cytotoxic subset of the natural killer cells. They are typically responsible for the direct killing of cancer cells through granzyme- and perforin-mediated mechanisms (79). However, in endometroid endometrial carcinoma (EEC), a distinct CD56dim_DNAJB1 NK cell subset was identified. These were seen to exhibit compromised cytotoxicity and elevated stress-related genes, including DNAJB1. The study also demonstrated that these abnormal NK cells engage vigorously with tumour-associated macrophages, fostering immune evasion in EEC. Further, it was observed that LAMP3^+^ dendritic cells inhibited CD8^+^ T-cell functionality and attracted regulatory T cells, supporting an immunosuppressive tumour microenvironment; collectively, the evidence presumes that therapeutic intervention against NK cell dysfunction and dendritic cell–mediated suppression may reconstitute antitumor immunity (77). Myeloid cells, especially dendritic cells and macrophages, act as pivotal regulators of the tumour-immune profile of the tumour’s niche. ScRNA-seq revealed that the overall abundance of macrophages was comparable in EC and normal endometrium but differed significantly in functional aspects. The tumour-biased CXCL8hi_IL1Bhi_Mac macrophages secreted proinflammatory and tumour-supporting cytokines like CXCL8, IL1B, and CCL3, inducing cancer cell growth, invasion, and metastasis. Conversely, the high abundance in normal tissues, C3_hi_IL1Blo_Mac, was associated with immune-regulatory gene expression such as C3 and CD1C, contributing to tissue homeostasis. The C1Qhi_IL1Blo_Mac subset occurred in both tissues but had more intense interactions with other immune cells in the tumour, indicating dynamic reprogramming of macrophage activities in the tumour microenvironment (80). Alongside molecular alterations, the tumour microenvironment is equally responsible for modulating the epithelial heterogeneity. Following the confirmation of EMT in EC, TTK, LY6K, and NOL4 have recently been reported as cancer-testis antigens with a bad prognosis, with TTK being particularly overexpressed in early-stage cancer. Single-cell RNA sequencing revealed a cryptic malignant proliferative subset co-expressing TTK-related genes and EMT markers, suggesting TTK-mediated EMT activation. This subset was further found to have high expression of UBE2C, which is a known p53 pathway regulator for EMT. Notably, this population’s gene signature was more evident in USC than in EEC and correlated with EMT-associated cells being associated with tumour aggressiveness and metastatic capability. These results underscore the potential for therapeutic intervention against EMT-associated pathways in EC (81).

In the majority of cancers, vimentin is an EMT marker and is associated with invasiveness and a worse prognosis (82, 83). Conversely, in a study, it was noticed that in EC at low stages, low epithelial expression of vimentin is associated with increased risk of recurrence and poorer survival. Through single-cell RNA analysis, the study failed to find significant differences in immunity or stroma between recurring and non-recurring tumours, but epithelial cell expression profiles—particularly vimentin—were related to prognosis. The results were confirmed across independent mRNA cohorts and IHC in 518 patients, making vimentin a tissue-specific prognostic biomarker. Hence, epithelial vimentin could therefore be used to identify low-stage patients at risk of recurrence and guide personalized treatment and follow-up (84). In a study, it was found that alterations in the population of some endometrial cells and gene expression patterns contribute to EC pathogenesis. Aberrant fibroblast, epithelial, and immune cell subpopulations showed significant transcriptional changes, indicating tumour progression and immune modulation. Downregulated were the critical genes CAV1, VWF, and DCN, suggesting loss of tumour-suppressive functions, and upregulated were SCGB2A1, CLDN4, and immune-related genes CCL3 and GZMB, promoting tumour growth and immune evasion. These findings suggest that disruption of normal endometrial cell communication and transcriptional regulation underlies EC development and may offer novel molecular targets for diagnosis and treatment (85). Diabetes has been associated with the incidence and prognostic projections of malignancies, notably EC. A study investigated the connection between Diabetes Mellitus (DM) and EC, focusing on the role of DM-associated genes in WFS1 in the alliance. scRNA-seq analysis with EC tissues revealed low expression of WFS1 in immune cells, especially monocytes. This was further validated by IHC. The WFS1 gene is present in the endoplasmic reticulum (ER) and is integral in modulating ER stress and glucose metabolism. These observations suggest that dysregulation of WFS1 may contribute to ER stress–induced tumour progression and could serve as a potential biomarker for poor prognosis in EC (86).

The upward trend of EC remains constant despite the implementation of targeted therapies and immunotherapies (87). Immune checkpoint molecules are generally self-tolerant but can paradoxically inhibit antitumour immunity within the TME by restricting immune-mediated tumour inhibition. Of these, PD-1 and CTLA-4 are the ones that are mostly studied. In mismatch repair-deficient (MMRd) endometrial cancer, elevated tumour mutation burden (TMB) theoretically facilitates immune recognition through presentation of neoantigen during immune checkpoint inhibition. scRNA-seq analysis of peripheral blood mononuclear cells identified unique immune signatures: mut-MMRd responders (mutR) had CD8+ T cells with KLRG1 and EOMES expression, while epiR had CD16+ NK cells with increased cytotoxic genes like GZMA and TRAIL, suggesting distinct mechanisms of antitumor immunity (51). Current studies have emphasized the significance of scRNA-seq in examining immune cell characteristics in low immune-responsive ECs, demonstrating enrichment for exhausted CD8+ T cells that bear inhibitory receptors (e.g. PD-1, CTLA-4, and LAG-3), in addition to higher regulatory T cells (Tregs) and myeloid-derived suppressor cells (MDSCs), which collectively play in a significant role in immunosuppression (52, 76, 77, 88). While the mechanisms driving the immune-related adverse events (irAEs) of immune checkpoint inhibitors (ICIs) remain unclear, evaluation of FAERS data indicated that PD-1 inhibitors initiate irAEs earlier than PD-L1 inhibitors. Subsequently, scRNA-seq unveiled PD-1–high CD8+ effector and Tfh cells strongly interacting with other TME cells through CXCL12-CXCR4 and CXCL16-CXCR6 pathways, which were not observed with PD-L1–high Tregs. This suggests that PD-1 blockade can induce acute irAEs by triggering compensatory activation of these chemokine axes. These observations underscore the value of profiling functional immune subsets and dynamic gene expression to enhance immunotherapy efficacy and direct combinatorial approaches (89).

## 4 Spatial transcriptomics

scRNA-seq has transformed transcriptomic profiling, but it poses critical limitations, including isolating viable cells from intact tissues, often inducing stress, cell death, or artificial aggregation, that can distort the biological environment (90). More importantly, cell dissociation eliminates spatial context, making it impossible to determine how cells interact within the naïve microenvironment. This lack of spatial information limits the understanding of how cellular organization drives tissue function and disease progression (91). In contrast, while IHC can reveal the location of proteins with high fidelity, it is typically constrained to a narrow panel of pre-defined markers and lacks the transcriptomic breadth necessary to map novel states or rare subpopulations. A comparison of the concerned methodologies, including IHC, scRNA-seq, and ST, has been tabulated in **Table 3**.

**Table 3:** Comparison of methodologies for elucidating tumour environment.

Features	Single-cell RNA seq	Spatial transcriptomics	Immunohistochemistry
Sample Collection Method and Processing	The sample collected is dissociated into single cells and then isolated and sequenced	The tissue sample is sectioned into thin slices and placed on specialized slides to capture the barcodes/probes	The tissue sample is fixed onto a slide and processed to visualize the desired protein
Resolution	Single-cell level	Spatial level visualization	Tissue and single-cell level
Spatial information	Lost	Preserved	Limited to selected markers
Molecular target	RNA	RNA	Protein
Throughput	High	Moderate	Low to moderate
Multiplexing Capability	High	High	Limited
Time consumption	High (Days to weeks)	Moderate to high (Days to weeks)	Low to moderate (Hours to few days)
Target Specificity	Whole transcriptome or targeted RNA panel	Whole transcriptome or targeted RNA panel	Specific protein detection
Cell Type Identification	High	Moderate, depending on spatial resolution	High, but limited to known cell markers
TME Heterogeneity Analysis	Identifies cellular population as well as subpopulations and their states	Captures the spatial organization of the cell types	Identifies the different cell types that express protein and their location
Expression profile	Provides gene expression at the single-cell level and identifies functional states of the cells	Reveals spatial gene expression	Provides functional insights at the protein level
Cost	High	High	Low
Clinical Application	Research-based potential for precision medicine, but limited use in clinical settings	Research-based potential for precision medicine, but limited use in clinical settings	Used for routine diagnostic and prognostic studies
Strengths	Identifies rare cell types, cellular states, and heterogeneity	Captures the cell-cell interaction in their spatial location	Direct visualization of specific protein localization and abundance
Limitation	Loss of spatial context	Lower spatial resolution, and often doesn’t reach the single-cell level	Restricted capacity to analyse multiple proteins simultaneously
Major Application	TME heterogeneity, cell-type identification, and trajectory analysis	TME Spatial organization and spatial gene expression	Biomarker validation

To overcome these challenges, Ståhl et al. (2016) introduced Spatial Transcriptomics (ST), a method that preserved spatial architecture while capturing gene expression patterns across the tissue slices (92). By retaining the positional information of transcripts, ST enables researchers to map the molecular landscape of tissues with spatial precision (12). This spatially resolved view is essential for deciphering cell-cell interactions that regulate critical processes such as tissue development, immune response and tumour evolution (93). Since its introduction, ST has been widely applied across multiple biological contexts, including central nervous system diseases (94), cardiovascular disease, chronic kidney disease (95). Alzheimer’s disease (96), skin pathologies (97) and importantly, cancer research (98).

**Figure 4:**
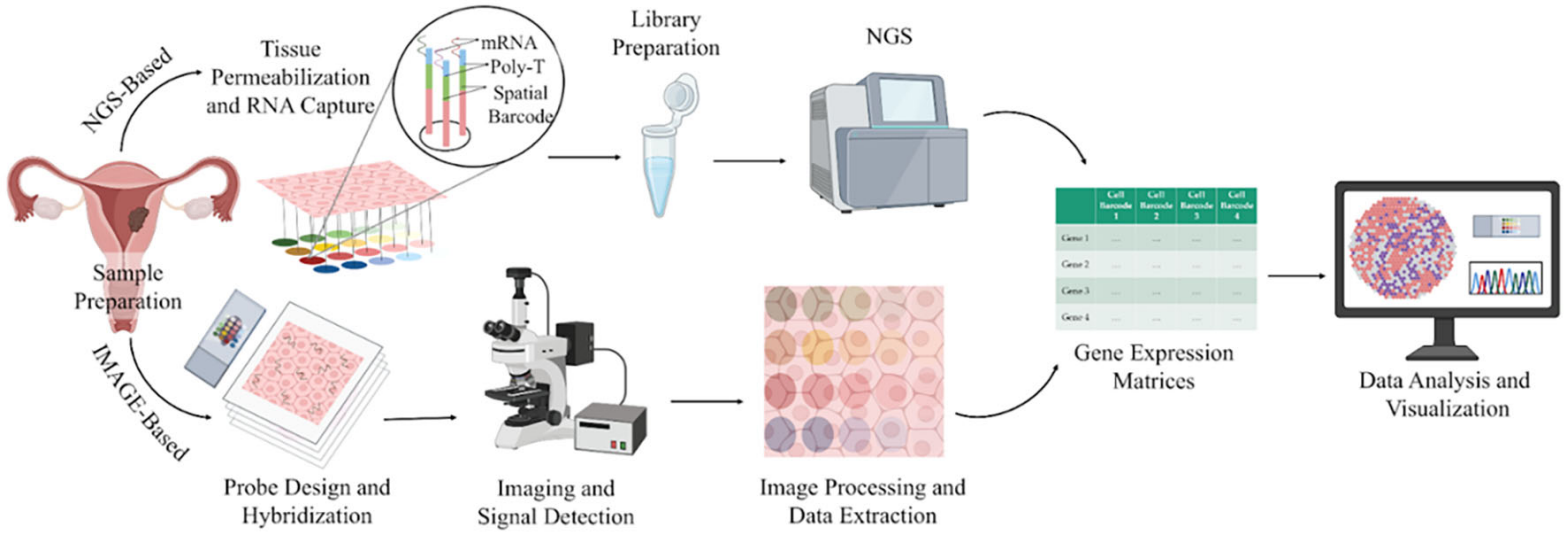
A schematic illustration of the spatial transcriptomics workflow. Image created by BioRender (13).

The analysis of ST data typically begins with pre-processing of either sequencing-based or image-based inputs to generate a gene expression matrix, which forms the foundation for all downstream bioinformatic analyses. Subsequent steps, such as batch correction, dimensionality reduction, spatial clustering, and cell-type annotation, ensure data accuracy and enable researchers to visualise the spatial distribution of transcriptional activity across tissues. Further analyses, including identification of patterns in gene expression and variable genes, spatial region classification, cell-cell interaction, spatial trajectory reconstruction, and exploration of gene-gene interactions, provide deeper insights into the organisation and functional architecture of the tissue (99). The complete workflow of ST is illustrated in **Figure 4**. Interestingly, integration of scRNA-seq with ST leverages the high-resolution transcriptomic map, thereby resolving complex TME (100). IHC complements these findings by validating the spatial functional identities of key cell populations at the protein level, ensuring translational relevance. Together, these methods yield a multidimensional view critical for understanding tissue biology in health and disease (101).

Despite these advantages, a few challenges also exist. The low cellular resolution in many ST platforms results in transcriptomic signals derived from clusters of multiple cells rather than true single cells, limiting cellular specificity. The high costs and technical complexity restrict the number of samples analysed, impacting large-scale cohort analyses. Critically, *in vitro* and *in vivo* validation of findings remains essential to confirm the functional relevance of spatially mapped gene expression patterns (102).

### 4.1 ST in endometrial cancer

Since ST primarily aids in identifying cellular populations in their respective spatial environment, a study was undertaken to identify the changes in the cellular populations within EZH2 conditional knockout mice and the wild-type mice. EZH2 aids in the regulation of chromatin condensation and inhibition of transcription. This conditional deletion demonstrates changes including uterine hypertrophy, cystic endometrial hyperplasia, epithelial hyperproliferation and E2 hyper-responsiveness. The ST analysis revealed distinct gene expression alterations localised specifically to the epithelial and stromal compartments. For instance, genes such as Asb4, Cxcl14, Dio2, and Igfbp5 were markedly upregulated within epithelial regions, while stromal domains exhibited increased expression of Cald1, Fbln1, Myh11, Acta2, and Tagln. This region-specific transcriptional remodelling highlighted a spatially coordinated dysregulation of signalling pathways driving epithelial proliferation and glandular hyperplasia (103). Furthermore, ST exploration in non-surgical treatments such as levonorgestrel intrauterine device (LIUD) therapy in atypical hyperplasia (AH) or grade 1 endometrioid endometrial cancer (G1EEC) demonstrated that LIUD increases the abundance of immune cells, particularly natural killer (NK) cells and cytotoxic lymphocytes with elevated levels of lymphocyte cytotoxicity markers. However, during relapse, the NK cells were seen to be decreased, whereas an escalation of immune exhaustion markers, such as IDO1, was observed. Interestingly, ST revealed that during relapse, immune pathways such as interferon-α and TGFβ signalling were seen to be enriched, suggesting that immune exhaustion and reversal of progestin-related immune modulation can contribute to relapse and treatment resistance (104).

**Figure 5:**
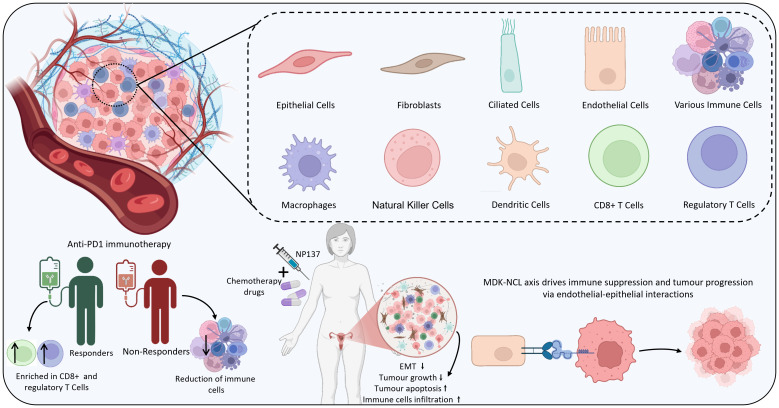
A schematic demonstration of findings of scRNA-seq and spatial transcriptomics.

Several studies emphasize integrating both scRNA-seq and ST to get a comprehensive view of the TME. A study investigating the heterogeneity of EC identified various cells, including epithelial cells, ciliated cells, endothelial cells, and immune cells, using scRNA-seq and ST. Furthermore, the results emphasized the communication between epithelial and endothelial cells mediated by the MDK (Midkine) and NCL (nucleolin) ligand-receptor pair that was found to promote malignant phenotypes and immune suppression in the TME. ST evidently highlighted that MDK was highly expressed predominantly in epithelial and ciliated cells and that the MDK-NCL interaction occurs in specific tissue regions, contributing to immune suppression and tumour progression (105). Furthermore, another study uncovered that employing ST, tumours could be stratified into hot, intermediate, and cold based on the immune profiles that are associated with CD8+ T cell infiltration. Further, potential biomarkers such as HLA class I, CD8, and DNMT3A were also identified, which assist in the prediction of immune checkpoint inhibitor therapeutic efficacy. These biomarkers were also validated using multiplexed immunofluorescence and immunohistochemistry (106). Interestingly, spatial transcriptomics also offers insights into the resistance or sensitivity mechanism of a treatment. For instance, a study on patients receiving anti-PD-1 immunotherapy unveiled that in the responders, CD8+ cytotoxic and regulatory T cells played key roles, whereas in non-responders, a reduction of immune activity was noticed. Additionally, the cell-cell communication within the TME of the responder was stronger than the non-responder. The ligand-receptor interactions, including CD74–APP, CD74– MIF, and CD74–COPA, were revealed to associate with CD8+ cytotoxic cells, regulatory T cells and others (107). Similarly, integrating ST with scRNA-seq and bulk RNA-seq across pre- and post-treatment samples aids in gaining comprehensive insights into the therapeutic mechanisms and potential predictors, paving the way for personalised treatments. ST, along with scRNA-seq, revealed that NP137, an anti-netrin-1 antibody, with carboplatin-paclitaxel helps in the reduction of epithelial-to-mesenchymal transition (EMT) conversion in EC. Also, the TME showed increased cytotoxic lymphocytes and antigen-presenting cells, whereas decreased cancer-associated fibroblasts and M2-like macrophages. Specifically, there was an increase in the strength of interaction and the number among the tumour and T cells. Altogether, these findings suggest that NP137 may enhance the tumour immune response, potentially through its influence on EMT. Given the growing evidence linking EMT to therapeutic resistance—particularly against chemotherapy and immune checkpoint inhibitors—the ability of NP137 treatment to suppress EMT-associated features highlights its promise for combination therapy (108). Findings from scRNA and ST are summarized in **Figure 5**. **Table 4** provides a summary of key findings from studies concerning IHC, single-cell, and spatial profiling approaches in EC. On the other hand, **Table 5** highlights the major applications of the three techniques.

**Table 4:** Summary of IHC, single-cell, and spatial transcriptomics studies with key findings.

Technique	Study focus	Sample/dataset	Key findings	Reference
IHC	Correlation between molecular subtypes and immune contexture in EC	142 therapy-naïve EC patients.	The study identified MSI tumours with MLH1/PMS2 defects, and high-grade POLE-wildtype/MSS tumours exhibited high immunogenicity, characterized by dense T-cell infiltration. Additionally, high Treg density was associated with a poor prognosis in p53-mutant EC.	(25)
	Exploration of the immune microenvironment	Patients with EC who underwent primary treatment	TLSs were present both intratumorally and in the stromal regions. It was composed of CD8^+^ T cells, CD4^+^ T cells, CD38^+^ plasma cells, and germinal centres, indicating an active immune microenvironment.	(37)
	Immune cell infiltration and TIL distribution	Patients with EC	TIL-rich tumours contained more CD8^+^ T cells, while immune-excluded ones had fewer CD8^+^ T cells.	(38)
	Prognostic significance of stromal TILs	30 histologically confirmed EC cases	High stromal TIL levels indicated a better prognosis in early-stage, low-grade EC.	(39)
	Immune checkpoint protein expression	EC tissues and blood samples from 99 patients	PD-L1 expression is associated with tumour immunogenicity; PD-1 is localized at the tumour–immune interface, suggesting a regulatory role in immune evasion.	(35)
	CXCR4 interaction with CAFs in TME	205 EEC patients	CXCR4 expression was found in the cytoplasm and cell membrane, linked with tumour invasion and interaction with CAF.	(41)
	Expression of GRHL1 and glutaminase	66 EC tissues and their surrounding normal tissues	Overexpression of GRHL1 and glutaminase correlated with tumour proliferation and metabolic remodelling.	(42)
	Heterogeneity of HER2 signalling in uterine serous carcinoma	Samples of endometrial serous carcinoma with documented HER2 status	HER2 showed an incomplete membranous and basolateral “U-shaped” staining pattern, revealing HER2 signalling heterogeneity.	(43, 44)
	Molecular profiling through cervical smear	Cervical smears in 50 patients with EC	Abnormal p53 and MSH2/MSH6 patterns with concurrent PTEN, ARID1A, PIK3CA, and TP53 gene changes suggest the feasibility of minimally invasive EC profiling.	(45)
scRNA-seq	Understanding immune cell characteristics in low-immune-responsive ECs	GSE173682, GSE225691, PRJNA650549, SRP349751, In-house dataset	Identified enrichment of exhausted CD8+ T cells expressing PD-1, CTLA-4, and LAG-3, alongside elevated Tregs and MDSCs, all contributing to strong immunosuppression.	(88)
	Cellular heterogeneity in EC	In-house dataset	Identified lymphoid, myeloid, and epithelial cell subtypes; The epithelial cells were further divided into stem-like, secretory glandular, and ciliated cells.	(78)
	NK cell and dendritic cell–mediated immune suppression in EEC	GSE173682, GSE225691, PRJNA650549, SRP349751	An abnormal CD56dim_DNAJB1 NK cell subset with reduced cytotoxicity and high stress responses interacting with tumour-associated macrophages, leading to immune evasion, was discovered. LAMP3+ dendritic cells inhibited CD8+ T-cell function and recruited Tregs, promoting an immunosuppressive TME.	(77)
	Gene expression and cell population changes driving EC pathogenesis	E-MTAB-10287	Transcriptional alterations in fibroblast, epithelial, and immune cells were found. Downregulated tumour suppressor genes (CAV1, VWF, DCN) and upregulated tumour-promoting/immune genes (SCGB2A1, CLDN4, CCL3, GZMB), indicating disrupted tumour suppression and enhanced immune evasion and tumour progression.	(85)
ST	Effects of EZH2 conditional knockout	EZH2 conditional knockout mice vs wild-type mice	Conditional deletion of EZH2 caused uterine hypertrophy, cystic endometrial hyperplasia, epithelial hyperproliferation, and E2 hyper-responsiveness. ST revealed compartment-specific gene expression in epithelial regions (Asb4, Cxcl14, Dio2, Igfbp5) and stromal regions (Cald1, Fbln1, Myh11, Acta2, Tagln). The spatially coordinated dysregulation also highlighted epithelial proliferation and glandular hyperplasia.	(103)
	ST in non-surgical treatment with LIUD	NCT00788671	LIUD increased NK cells and cytotoxic lymphocytes with elevated cytotoxicity markers; relapse was linked to decreased NK cells, increased immune exhaustion markers (IDO1), and enriched interferon-α and TGFβ signalling, suggesting immune exhaustion and resistance.	(104)
	Tumour immune profiling	In-house dataset	Tumours stratified as hot, intermediate, or cold based on CD8+ T cell infiltration; identified biomarkers (HLA class I, CD8, DNMT3A) predictive of immune checkpoint inhibitor efficacy; biomarkers validated via multiplexed immunofluorescence and immunohistochemistry.	(106)
scRNA-seq and ST	Access the heterogeneity of EC TME	GSE173682	Identified epithelial, ciliated, endothelial, and immune cells; communication between epithelial and endothelial cells mediated by MDK–NCL ligand-receptor pair, promoting malignant phenotype and immune suppression.	(105)
	Response to anti-PD-1 immunotherapy	GSE251923	In responders, CD8+ cytotoxic and regulatory T cells were key; non-responders showed reduced immune activity, and also stronger cell-cell communication in responders.	(107)
	Effect of NP137 with carboplatin-paclitaxel in EC	GSE225691	NP137 suppressed EMT, increased cytotoxic lymphocytes and antigen-presenting cells, decreased CAFs and M2-like macrophages; increased tumour–T cell interactions; suggests NP137 enhances tumour immune response and may benefit combination therapy.	(108)

**Table 5:** Applications of IHC, scRNA-seq, and spatial transcriptomics in EC.

Technology	Applications in endometrial carcinoma
Immunohistochemistry	• Diagnostic classification and subtyping • Prognosiss and risk prediction • Guides treatment decisions, including immunotherapy
Single-cell RNA sequencing	• Profiles gene expression at single-cell resolution, validating expression at the cellular level versus bulk RNA-seq, which provides averaged expression across mixed populations • Identifies heterogeneous cellular populations • Maps cell-cell interactions, revealing communication between neighbouring cells, influencing tumour progression • Reveals proportions and states of immune and stromal cells, distinguishing immune-active versus immune-suppressed TME • Uncovers mechanisms underlying immune resistance and aids in precision medicine
Spatial transcriptomics	• Identifies cell populations in their respective spatial environment • Allows gene expression analysis in different cells, similar to scRNA-seq, but within tissue context • Reveals region-specific transcriptional remodelling • Maps cell-cell interactions between neighbouring cells • Identifies ligand-receptor pairs that can promote tumour progression or drive immune suppression, such as MDK-NCL • Stratifies tumours based on CD8+ T cell infiltration patterns • Offers insight into mechanisms of tumour resistance and sensitivity to treatments

## 5 Conclusion and future perspective

Understanding and deciphering the TME of endometrial cancer has been crucial for advancing the diagnosis and treatment options. The FIGO 2023 grading system has modernized EC staging by incorporating molecular classification and refined histological criteria, leading to a more precise risk stratification and tailored treatment strategies. This update also enhances prognostic accuracy and clinical management by reflecting the biological diversity of tumours better than previous systems. The various studies of single-cell RNA sequencing, spatial transcriptomics, and immunohistochemistry have markedly advanced our understanding of EC TME, heterogeneity, and immune infiltration. These techniques have also aided in understanding the different molecular classes and immune profiles that unveil the biology of EC.

IHC remains an essential tool to validate molecular findings and guide clinical diagnostic workflows, while scRNA-seq has identified diverse immune and epithelial cell states, highlighting potential therapeutic targets. Spatial transcriptomics has provided crucial spatial context to these cellular interactions, enhancing biomarker identification for patient stratification. Moreover, these approaches pave the way toward personalized treatment strategies by gaining a comprehensive view into the mechanisms of treatments, their role in causing resistance, and immune landscape characterization. Collectively, these new molecular insights help in refining the diagnostic approaches, prognostic tools, and can also be utilized as predictive tools for patient stratification for therapies, including immunotherapy.

However, certain potential limitations need to be investigated. IHC, though cost-efficient, offers only a limited snapshot of the TME, potentially overlooking key cellular interactions and heterogeneity. These constraints are overcome by scRNA-seq, which provides the complete picture of the tumour microenvironment and uncovers complex cell-cell interactions. Nonetheless, scRNA-seq fails to maintain the spatial integrity within the tissue. This shortcoming is overcome by ST, which preserves tissue architecture and allows visualization of cellular organization *in situ*. Despite its advantages, the broader adoption of ST remains restricted due to its requirement for specialized equipment, advanced analytical expertise, and high operational costs, which can limit accessibility and scalability. Nevertheless, ongoing technological improvements and cost reductions are expected to broaden their application.

Further research should focus on integrating artificial intelligence, machine learning, and deep learning approaches for predictive modelling and integrating various other omics into single and spatial transcriptomics. By bridging these gaps, these fundamental discoveries can be translated into effective clinical practices to improve patient outcomes and precision treatments. Hence, the continued integration of IHC, scRNA-seq, and ST in EC research and clinical practice will be crucial in deepening our understanding of the disease mechanisms, gaining insights into the molecular classifications, and guiding the evolution of a personalized treatment paradigm.
